# Human adipose-derived stem cell adipogenesis induces paracrine regulation of the invasive ability of MCF-7 human breast cancer cells *in vitro*

**DOI:** 10.3892/etm.2013.1237

**Published:** 2013-07-30

**Authors:** YANG ZHAO, JIANHUA GAO, FENG LU

**Affiliations:** Department of Plastic Surgery, Nanfang Hospital, Southern Medical University, Guangzhou, Guangdong 510515, P.R. China

**Keywords:** human adipose-derived stem cells, human breast cancer cells, paracrine, invasive, cytokine, metalloproteinase

## Abstract

The aim of this study was to determine the effects of paracrine regulation on the invasive ability of MCF-7 human breast cancer cells through human adipose-derived stem cell (hADSC) adipogenesis. hADSC differentiation of the third and fourth passages of cells was induced in different induction media: osteogenic, adipogenic and chondrogenic. Transwell migration assays in the differently conditioned media, flow cytometry, enzyme-linked immunosorbent assay and western blot analysis for selected cytokines were performed. The flow cytometric analysis demonstrated positive expression of CD29, CD44 and CD105, while expression of CD34 and CD45 was not identified. The transwell migration assay showed that the invasive ability of MCF-7 cells was significantly enhanced during hADSC adipogenesis. hADSCs exerted a significantly positive effect on the invasive activity of MCF-7 cells during adipo-genesis. The results indicate that the high expression levels of activating protein 2 (aP2) in MCF-7 and adipocytes induced for 12 days may be associated with cell growth, invasion and metastasis. Peroxisome proliferator-activated receptor γ may be involved in fatty syntheses during adipogenic initiation and following adipogenic differentiation, possibly acting as a protection factor resulting in cell maturation and differentiation. This study also demonstrated that the expression of vascular endothelial growth factor was repressed by hADSCs, while that of matrix metalloproteinase-2 and urokinase-type plasminogen activator was increased to a significant level.

## Introduction

Fat transplantation is extensively applied in cosmetic and reconstructive surgery as fat is an autologous material, with specific applications in mammaplasty, such as for treating breast deformity and asymmetry (1,2). At present, breast cancer is the second most common type of cancer and the preferred treatment is surgical therapy (3). However, conservative surgery that treats breast cancer by local excision has been demonstrated to be associated with anxiety and depression in patients with breast cancer and secondary breast deformity. In such cases, autologous fat grafting enables repair and augmentation of soft tissues, with the advantages of biocompatibility, versatility, natural appearance and low donor site morbidity (4). Although recovery of the fat implant and hyperplasia of adipocytes (ACs) are directly associated with human adipose-derived stem cells (hADSCs) (2,5), whether fat granule transplantation may be applied to patients following mammary cancer surgery and whether hADSCs enhance the growth and invasive ability of residual cancer remain unknown.

The transcription factors peroxisome proliferator-activated receptor γ (PPARγ) and activating protein 2 (aP2), are involved in the adipogenic differentiation of hADSCs and the occurrence, progression and prognosis of cancer (6–8). PPARγ is expressed in breast, pancreatic, testicular and other types of tumor cells (9). The low level of PPARγ expression observed in breast cancer tissue has been suggested to be a possible therapeutic target for the prevention of breast cancer progression (10). A previous study demonstrated that the activation of PPARγ is able to inhibit cancer cell growth (11), and this may be due to the inhibition of angiogenesis (12–14). aP2 is critical for regulating gene expression during early development and in breast cancer (15).

Therefore, the present study investigated the dynamic behavior of relevant transcription factors and the paracrine effects on MCF-7 human breast cancer cells during hADSC adipogenesis, providing a basis for the clinical application of fat transplantation.

## Materials and methods

### Cell culture and conditioned media

Adipose tissue samples were obtained from the subcutaneous abdominal fat tissues of three patients (age, 2–4 years) from the Department of Plastic Surgery of Nanfang Hospital (Guangzhou, China) who had been submitted for surgical treatment of a cicatrix by dermoplasty with a full-thickness skin graft. Patients with inflammatory or malignant diseases were not included in the study. This study was approved by the Research Ethics Committee of Southern Medical University (Guangzhou, China). All guardians provided informed consent.

Adipose tissue was obtained from the subcutaneous fat tissue at the donor site of a full-thickness skin graft taken during the dermoplastic treatment of a cicatrix. Cell isolation and culture were performed according to the method described by Zuk *et al* (16) with certain modifications. Briefly, following the removal of all fibrous material and visible blood vessels, adipose tissue samples were cut into small pieces (10–15 mm) and digested in 10 mM phosphate-buffered saline (PBS; Sigma, St. Louis, MO, USA) containing 0.75% collagenase I (Sigma) for 30–60 min in a shaking water bath at 37°C. The dispersed material was centrifuged (170 × g, 25°C) for 5 min, and the pellet was resuspended and seeded in flasks. After 24 h, the medium was replaced with fresh medium.

Cells were cultured for up to three to four passages in triplicate. For each passage, 1×10^6^ cells were seeded in 75-cm^2^ culture flasks for 7–10 days. When the cells attached to the flask reached ∼80% confluence, subculture (passage) was performed by enzymatic digestion (0.25% trypsinization). Cells in the second to fourth passage were used for experiments. MCF-7 cells were obtained from the Cell Bank at the Chinese Academy of Sciences (Shanghai, China). Cell lines used in this study were maintained in a humidified (5% CO_2_) incubator. Cells were cultured in Dulbecco’s modified Eagle’s medium (DMEM) containing 10% fetal bovine serum (FBS; Gibco-BRL, Carlsbad, CA, USA) and 2 mM L-glutamine (Gibco-BRL).

### Expansion and differentiation of hADSCs

The hADSCs were seeded by suspending 2–3×10^5^ cells/ml in 24-well plates, and cultured in osteogenic, adipogenic or chondrogenic induction medium (Table I). The medium was replaced every 3 days. Adipocytes induced for 6 or 12 days were termed as the AC-6d and AC-12d groups, respectively.

### Immunohistochemical staining

Cells were cultured and fixed after 14 days. ACs were identified as red lipid droplets upon staining with Oil Red O. Differentiated osteogenic cells were stained with Alizarin Red-S, alkaline phosphatase and Von Kasso. All staining markers were purchased from (Genmed Scientifics Inc., Arlington, MA, USA).

### Flow cytometric analysis

Cell aliquots (2×10^6^ cells/ml) were incubated with monoclonal antibodies (Caltag, Carlsbad, CA, USA): fluorescein isothiocyanate (FITC)-conjugated anti-human-CD29, -CD34, -CD44, -CD45 and -CD105, respectively, for 30 min, and washed with PBS prior to analysis.

### Cell invasion assay

For the invasion assay, 2.5×10^5^ MCF-7 cells were seeded in the upper well of each transwell chamber (Corning Inc., Tewksbury, MA, USA). Conditioned culture medium (300 *μ*l; Table I) and 300*μ*l DMEM containing 20% FBS were placed in the lower compartment of the chemo-taxis chamber as a source of chemoattractants. Cells were incubated for 24 h at 37°C with 5% CO_2_. Cells that had invaded the lower surface of the membrane were fixed with methanol and stained with hexamethylpararosaniline (GenMed). Using light microscopy, at least four random fields were selected and the cells in each field were counted. Subsequently, the cells were eluted in 600 *μ*l 33% acetic acid (Shanghai Sangon Biological Engineering Technology and Services Co., Ltd., SongJiang, China) for 10 min and the optical density (OD) of the final cells through the matrigel (R&D Systems, Minneapolis, MN, USA) was determined at 570 nm. The MCF-7 cells grown in standard medium were set as the control group.

### Cytokine measurement

The concentrations of vascular endothelial growth factor (VEGF), matrix metalloproteinase 2 (MMP-2) and MMP-9 were detected with Quantikine ELISA kits (R&D systems, Minneapolis, MN, USA). The concentrations of urokinase-type plasminogen activator (uPA) were measured with uPA Activity Assay kit (EMD Millipore Corporation, Billerica, MA, USA. The OD was read using a spectrophotometer set at a wavelength of 450 nm within 30 min.

### Western blot analysis

Cells were centrifuged at 240 × g for 1 min at room temperature. Following centrifugation, cells were washed twice with PBS, and the supernatant containing proteins was extracted and quantified on ice. Each lane was loaded with samples on 8 and 12% vertical sodium dodecyl sulfate-polyacrylamide gel electrophoresis gel, separated and electro-transferred to polyvinylidene fluoride ultrafiltration membranes (Millipore, Billerica, MA, USA). The primary antibodies against aP2 (1:200), PPARγ (1:200), and GAPDH (1:1,000), as well as the secondary antibodies (1:10,000), were purchased from Santa Cruz Biotechnology Inc. (Santa Cruz, CA, USA).

### Statistical analysis

Protein levels were determined by western blot analysis with Quantity One software (Bio-Rad, Hercules, CA, USA). SPSS software, version 13.0 (SPSS, Inc., Chicago, IL, USA) was used for the Student’s unpaired t-test and rank-sum test. Data are expressed as the mean ± standard deviation (SD). P≤0.05 was considered to indicate a statistically significant difference.

## Results

### Characterization of differentiated hADSCs

**Figure 1.**
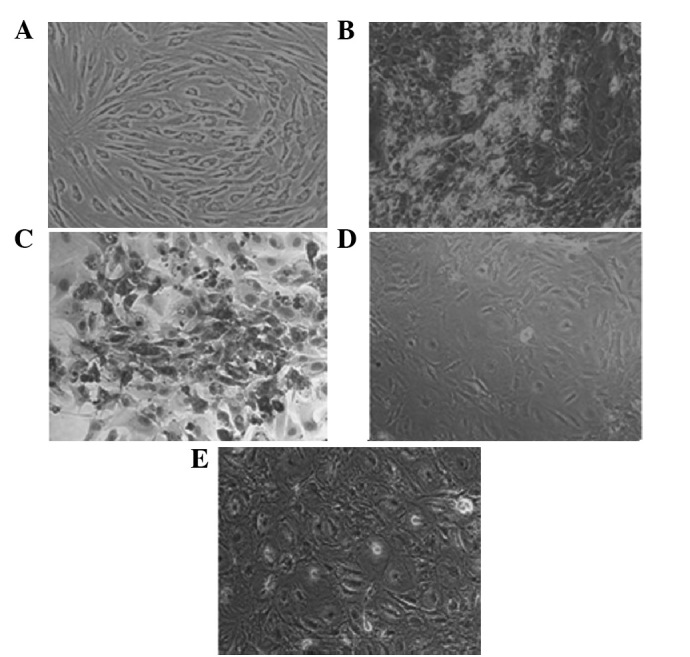
Adipogenic and osteoblastic differentiation induced by human adipose-derived stem cells (hADSCs). (A) Morphological observation of hADSCs under an inverted light microscope (magnification, ×40); (B) light microscopy observation of adipogenic differentiation on day 11 (magnification, ×40); (C) osteoblastic differentiation (magnification, ×40); (D) Oil Red O staining of adipogenic differentiation on day 12 (magnification, ×100) and (E) Alizarin Red-S staining of osteoblastic differentiation (magnification, ×40).

**Figure 2.**
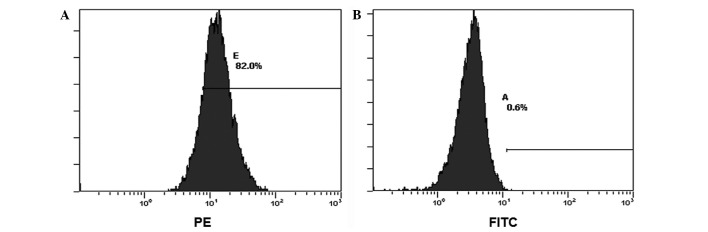
Positive and negative surface markers assessed by flow cytometry. (A) The presence of CD29, CD44 and CD105 in the third generation human adipose-derived stem cells (hADSCs) was ∼82%, while that of (B) CD34 and CD45 was 0.6%.

To confirm that the culture-expanded cells were true stem cells, the original phenotype and mesodermal differentiation potential upon exposure to chondrogenic, osteogenic and adipogenic specific agents were examined. Compared with the third generation of hADSCs (Fig. 1A), the presence of lipid droplets characteristic of adipogenic cells (Fig. 1B), and calcium deposits characteristic of osteogenic cells (Fig. 1C) was observed, and was confirmed by Oil Red O and Alizarin Red-S staining, respectively (Fig. 1D and E). Flow cytometry analysis revealed expression of CD29, CD44 and CD105, but no expression of CD34 and CD45 in the third generation (Fig. 2). In conclusion, these results indicated that the expanded cells possessed the basic properties of differentiation.

### Invasion ability assay of MCF-7 in vitro

**Figure 3.**
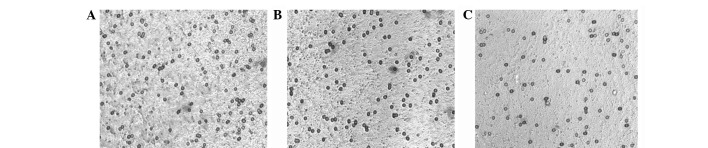
Morphological features of MCF-7 in differently conditioned media by transwell migration assay. MCF-7 invasion of human adipose-derived stem cells (hADSCs) in (A) conditioned medium, (B) adipogenically conditioned medium and (C) 10% fetal bovine serum standard medium. Magnification, ×80.

A transwell assay was performed to evaluate the invasion activity of MCF-7 cells. Morphological invasive features of MCF-7 in the differently conditioned media are shown in Fig. 3. MCF-7 invasion was markedly enhanced by the inductive effects of ACs (Table II) for 12 days (AC-12d) and a greater invasive MCF-7 migration through the matrigel was detected compared with that of the control group. Moreover, the conditioned media for hADSCs and adipogenic induction both increased the level of MCF-7 invasion to a significant level (Table II). These results suggest that hADSCs enhance the invasive activity of MCF-7 cells.

### Expression of PPARγ and aP2

**Figure 4.**
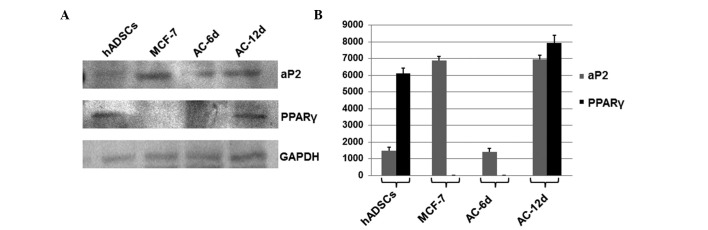
Expression of peroxisome proliferator-activated receptor γ (PPARγ) and activating protein-2 (aP2) in MCF-7, human adipose-derived stem cells (hADSCs) and adipocytes induced for 6 or 12 days (AC-6d and AC-12d, respectively). (A) Western blot analysis of aP2, PPARγ and glyceraldehyde 3-phosphate dehydrogenase (GAPDH) expression. GAPDH served as a loading control. (B) Comparison of the expression levels of aP2 and PPARγ.

To investigate the dynamic behavior of transcription factors associated with paracrine regulation, the expression levels of PPARγ and aP2 in MCF-7 cells, hADSCs, and AC-6d and AC-12d group cells during the entire adipogenic process were analyzed by western blotting. GAPDH served as a loading control. As shown in Fig. 4, no significant differences were observed in aP2 expression between the hADSC and AC-6 d groups; however, a significant increase in the AC-12 d group was observed when compared with that of the hADSC group (P<0.05). This indicated that the increased expression of aP2 was accompanied by adipogenic differentiation. Therefore, this suggests that the high expression level of aP2 in MCF-7 and the AC-12 d group may be closely associated with cell growth, invasion and metastasis.

No significant differences in the PPARγ expression level were detected between the hADSCs and AC-12 d groups, and no expression was found in the AC-6 d group and MCF-7 cells. The absence of PPARγ indicates that it may be associated with fatty synthesis during adipogenic initiation and following adipogenic differentiation, and may possibly act as a protection factor resulting in cell maturation and differentiation.

### ELISA measurements for selected cytokines

To evaluate the paracrine effects on secreted cytokines, the VEGF, MMP-2, MMP-9 and uPA levels were determined in the differently conditioned media. As shown in Table III, the VEFG concentration in the hADSC induction group was markedly lower than that in the control and AC induction groups. However, no significant difference was identified between the control and the AC induction groups. The MMP-2 level in the control group was too low to be detected, and no significant difference was observed between the MMP-2 levels in the hADSC and AC induction groups. By contrast, the MMP-9 level in the hADSC induction group was markedly higher than that of the AC induction group and marginally higher than that of the control group. The concentration of uPA in the hADSC induction group was similar to that in the AC induction group and the two groups showed a significantly higher concentration of uPA in comparison with the control group. These results indicate that VEGF expression is markedly inhibited by hADSCs, while the expression levels of MMP-2 and uPA are increased to a significant extent.

## Discussion

In this study, the hADSCs of the third generation showed significantly higher chemotaxis and invasive effects on MCF-7 cells than the cells treated with adipogenic induction. Breast cancer cells have been demonstrated to be closely associated with the expression of uPA, MMP-2, MMP-9 and VEGF (17,18). uPA enables extracellular matrix degradation by catalyzing the metalloproteinase precursors to activate MMP-9 (19,20) and enhancing endotheliocyte proliferation for blood vessel formation. VEGF may also be activated by uPA, allowing the infiltration and proliferation of cancer cells (21,22). In the present study, ELISA demonstrated that the levels of uPA were parallel with those of VEGF in breast cancer cells. The uPA levels in the hADSC and AC induction groups were lower compared with those of the control; however, the invasive ability of MCF-7 remained significantly increased under the same condition, which may be explained by the functional overlap of uPA. Therefore, it may be possible that the improvement of tumor invasion and metastasis occurs during fat implantation. Notably, compared with hADSC induction, the secreted VEGF level was higher in the adipogenic induction, which may be due to vasoformation during adipose tissue growth in the late adipogenesis stage.

As members of the metalloproteinase family, MMP-2 and -9 are associated with breast cancer invasion and metastasis, as well as bone destruction (23,27). MMP-2 is associated with local infiltration, while MMP-9 is the main participant in cancer recurrence and metastasis (19,25). The present study demonstrated that the levels of MMP-2 and MMP-9 in the hADSC and adipogenic induction groups were different from each other. The higher expression of MMP-9 in the hADSCs suggests a strong capability for matrix degradation. During this period, the presence of hADSCs enables growth and expansion at an early stage following fat implantation. Shortly after two weeks of adipogenic induction, subsequent adipogenesis occurs, resulting in a reduction of the level of MMP-9 and reduced expansion in the late stage following fat implantation. Therefore, recurrence and metastasis requires increased attention at the early stage, in which period the effect of hADSCs on breast cancer cells mainly occurs. Additionally, the increased level of MMP-2 in the hADSC and AC induction groups, which is consistent with the results from the transwell assay, provides further support for the theory that MMP-2 is closely associated with local infiltration.

The current study demonstrated that the level of PPARγ was higher in the hADSCs and 12 days following adipogenic differentiation, but that PPARγ was not expressed in MCF-7 cells or 6 days following adipogenic differentiation. This difference indicates that PPARγ is mainly involved in the early and midterm stages of the adipogenic differentiation of hADSCs, which is in accordance with a previous study whereby PPARγ regulated adipogenesis initiation (26). However, the inhibitory effect of PPARγ on breast cancer cells was weakened in the midterm stage. In the late-stage adipogenic differentiation of hADSCs, the level of VEGF was increased along with an increase in the level of PPARγ, which may be regulated by other pathways involved in adipocyte growth and angiogenesis.

## Figures and Tables

**Table I.** Components of the culture medium.

Induction type	Components	Concentration
Osteogenic	DMEM	-
	FBS	10%
	Dexamethasone	0.1 *μ*M
	β-glycerophosphate disodium	10 mM
	Vitamin C	50 *μ*g/ml
Adipogenic	DMEM	-
	FBS	8%
	Dexamethasone	1 *μ*M
	Insulin	10 *μ*M
	Indomethacin	200 *μ*M
	Isobutyl methyl-xanthine	0.5 mM
Chondrogenic	FBS	1%
	TGF-β1	10 ng/ml
	Insulin	6.25 *μ*g/ml
	Siderophilin	6.25 *μ*g/ml
	Dexamethasone	0.1 *μ*M
	Vitamin C	50 *μ*g/ml

DMEM, Dulbecco’s modified Eagle’s medium; FBS, fetal bovine serum; TGF-β1, transforming growth factor-β1.

**Table II.** Detected MCF-7 cell numbers at 450 nm in the presence of differently conditioned media.

Group	OD
hADSC-induced	0.263±0.009^a^
AC-12d-induced	0.202±0.004^a^
Control	0.184±0.003

a P<0.01, compared with the control group.

hADSC, human adi-pose-derived stem cell; AC, adipocyte; OD, optical density.

**Table III.** Concentrations of VEGF, MMP-2, MMP-9 and uPA under differently conditioned media by ELISA analyses.

Group	VEGF (pg/ml)	MMP-2 (pg/ml)	MMP-9 (pg/ml)	uPA (pg/ml)
hADSC-induced	187.450±20.61^a^	(4.77×10^4^)±30^a^	(1.930×10^3^)±190.00	(4.80×10^3^)±266.67^a^
AC-12d-induced	278.970±56.89	(4.93×10^4^)±22^a^	(0.618×10^3^)±156.00^a^	(5.10×10^3^)±91.30^a^
Control	320.945±28.03	0	(1.370×10^3^)±186.67	(1.70×10^4^)±566.67

a P<0.01, compared with the control group.

VEGF, vascular endothelial growth factor; MMP, matrix metalloproteinase; uPA, urokinase-type plasminogen activator; ELISA, enzyme-linked immunosorbent assay; hADSC, human adipose-derived stem cell; AC, adipocyte.
